# Dynamics of Envelope Evolution in Clade C SHIV-Infected Pig-Tailed Macaques during Disease Progression Analyzed by Ultra-Deep Pyrosequencing

**DOI:** 10.1371/journal.pone.0032827

**Published:** 2012-03-12

**Authors:** For Yue Tso, Damien C. Tully, Sandra Gonzalez, Christopher Quince, On Ho, Patricia Polacino, Ruth M. Ruprecht, Shiu-Lok Hu, Charles Wood

**Affiliations:** 1 Nebraska Center for Virology and the School of Biological Sciences, University of Nebraska-Lincoln, Lincoln, Nebraska, United States of America; 2 Department of Pharmaceutics, University of Washington, Seattle, Washington, United States of America; 3 The Washington National Primate Research Center, University of Washington, Seattle, Washington, United States of America; 4 Dana-Farber Cancer Institute, Boston, Massachusetts, United States of America; 5 Harvard Medical School, Boston, Massachusetts, United States of America; 6 School of Engineering, University of Glasgow, Glasgow, Scotland, United Kingdom; Commissariat a l'Energie Atomique(cea), France

## Abstract

Understanding the evolution of the human immunodeficiency virus type 1 (HIV-1) envelope during disease progression can provide tremendous insights for vaccine development, and simian-human immunodeficiency virus (SHIV) infection of non-human primate provides an ideal platform for such studies. A newly developed clade C SHIV, SHIV-1157ipd3N4, which was able to infect rhesus macaques, closely resembled primary HIV-1 in transmission and pathogenesis, was used to infect several pig-tailed macaques. One of the infected animals subsequently progressed to AIDS, whereas one remained a non-progressor. The viral envelope evolution in the infected animals during disease progression was analyzed by a bioinformatics approach using ultra-deep pyrosequencing. Our results showed substantial envelope variations emerging in the progressor animal after the onset of AIDS. These envelope variations impacted the length of the variable loops and charges of different envelope regions. Additionally, multiple mutations were located at the CD4 and CCR5 binding sites, potentially affecting receptor binding affinity, viral fitness and they might be selected at late stages of disease. More importantly, these envelope mutations are not random since they had repeatedly been observed in a rhesus macaque and a human infant infected by either SHIV or HIV-1, respectively, carrying the parental envelope of the infectious molecular clone SHIV-1157ipd3N4. Moreover, similar mutations were also observed from other studies on different clades of envelopes regardless of the host species. These recurring mutations in different envelopes suggest that there may be a common evolutionary pattern and selection pathway for the HIV-1 envelope during disease progression.

## Introduction

The envelope gene of human immunodeficiency virus type 1 (HIV-1) is the most genetically diverse among all HIV-1 genes. The vital role of HIV-1 envelope in determining cell tropism of the virus and escape from host immune surveillance made it a logical choice as the main focus for vaccine development. Thus, a better understanding of how the envelope evolves during disease progression could aid in designing better vaccines. Several envelope mutations, such as increases in the length of V1V2 variable loops and number of potential N-glycosylation sites (PNGS), have been linked with disease progression in humans [1], [2], [3]. Since these mutations were observed in envelopes from different clades, it would suggest that the envelope might tend to follow a certain evolutionary pattern during disease progression. Infection of non-human primates with simian-human immunodeficiency virus (SHIV) would be an ideal platform for investigating such HIV-1 envelope evolution during disease progression.

SHIV strains have been a significant tool in studying the role of HIV-1 envelope in pathogenesis and the development of AIDS vaccines for over a decade. Since their inception, SHIV constructs have undergone dramatic improvements to recapitulate many of the features of primary HIV-1 infection when used to infect rhesus macaques. One such design, SHIV-1157ipd3N4, expresses an R5 tropic HIV-1 clade C envelope isolated from a Zambian infant [4]. In addition, SHIV-1157ipd3N4 is pathogenic and fully capable of mucosal transmission through multiple routes [4], [5]. These properties closely resemble those of recently transmitted HIV-1 isolates, which are mostly R5 tropic and transmitted via mucosal routes [6], [7], [8], [9]. The fact that SHIV-1157ipd3N4 carries an HIV-1 clade C envelope makes this SHIV an important model to study transmission and pathogenesis of HIV-1 infection in humans: because more than fifty percent of all HIV-1 infections worldwide are caused by HIV-1 clade C [10], [11].

Until recently, SHIV-1157ipd3N4 had only been utilized to infect rhesus macaques (*Macaca mulatta*), a popular animal model for studying HIV pathogenesis and AIDS vaccine development. However, there is now a heightened interest in the pig-tailed macaque (*Macaca nemestrina*) model. In comparison to rhesus macaques, pig-tailed macaques are unique as they express a defective host restriction factor TRIM5α and hence are susceptible to infection by simian-tropic HIV-1 strains [12], [13], [14], [15]. Given this recent focus on the pig-tailed macaque animal model, it will be important to examine the relationship between disease progression and envelope evolution in pig-tailed macaques infected by SHIV-1157ipd3N4. Moreover, this study will help to determine whether envelope changes observed in a human and in a rhesus macaque during disease progression are present in this pig-tailed model. In an earlier report, Ho et al. demonstrated that SHIV-1157ipd3N4 was able to infect pig-tailed macaques by intrarectal inoculation and to cause AIDS in the infected animal [16]. We procured samples from these infected pig-tailed macaques and employed 454 ultra-deep pyrosequencing (UDPS) to characterize the HIV-1 clade C envelope changes as the infected animal progressed to AIDS.

UDPS is a high-throughput sequencing technology that can rapidly generate a vast amount of sequencing data in a cost-effective manner. The high sensitivity of this technology in detecting minor populations makes it an ideal approach for our study. UDPS has been used by several groups to study minor drug resistance mutations, cytotoxic T-lymphocytes (CTL) escape pathways and small envelope region such as V3 [17], [18], [19], [20], [21], [22]. To our knowledge, this is the first study to use UDPS to resolve HIV-1 clade C envelope evolution in infected pig-tailed macaques during disease progression which is marked by a consistent decline in CD4^+^ T-cell count and the present of high plasma viral load. AIDS is established when the CD4^+^ T-cell count falls <200 cells/ul. By using UDPS, we were able to quantitatively assess the emergence and outgrowth of minor variants in the infected animals as the disease progressed and provide a detailed genetic analysis of such variants. Interestingly, we found several gp120 mutations that evolved in parallel with disease progression. In addition, we compared the mutations observed in the pig-tailed macaque with published data on the evolution of the precursor envelope of SHIV-1157ipd3N4 in a rhesus macaque and the Zambian infant, from whom the viral envelope was originally isolated [23], [24]. Our analysis showed that several shared gp120 mutations were prevalent in the infected human, rhesus and pig-tailed macaques throughout disease progression. This study suggests that the HIV-1 clade C envelope may follow a comparable evolutionary pattern and host selective pressure during disease progression in infected hosts, regardless of the host species.

## Results

### Infection of pig-tailed macaques with SHIV-1157ipd3N4

**Figure 1:**
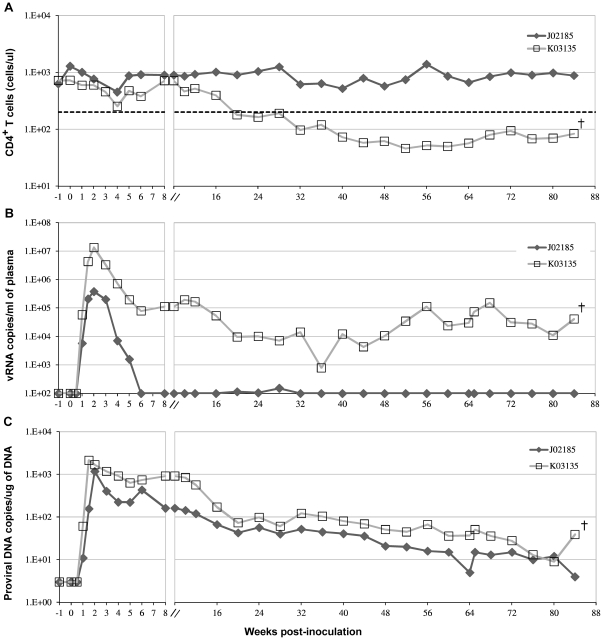
CD4^+^ T-cell counts, plasma and PBMC viral loads from infected pig-tailed macaques. (A) Total CD4^+^ T-cell counts (B) viral RNA loads in plasma and (C) proviral cDNA loads in PBMC. A scale break (//) representing 8 weeks post-inoculation was insert into the x-axis to show the early stage of infection. A cross (+) represents death of the animal due to AIDS.

Detailed examination of the immunological responses of the infected pig-tailed macaques has been conducted previously [16]. Briefly, after intrarectal inoculation of SHIV-1157ipd3N4, systemic infection was achieved in all four juvenile pig-tailed macaques with an average peak plasma viral RNA load >7×10^6^ copies/ml by 2 weeks post-inoculation (Fig. 1B). However, two infected animals (M04123 and L03165) died due to unrelated causes during sampling procedures at 2 and 48 weeks post-inoculation, respectively (data not shown). The remaining animals (K03135 and J02185) were monitored over a period of 84 weeks. The plasma viral loads of macaque K03135 increased while its CD4^+^ T-cell counts declined as disease progressed (Fig. 1A and B). Macaque K03135 developed AIDS with a CD4^+^ T-cell count consistently <200 cells/ul from 20 weeks post-inoculation onwards. Therefore, macaque K03135 was classified as a progressor. Meanwhile, macaque J02185 had normal CD4^+^ T-cell counts over the entire study period (Fig. 1A). Plasma viral loads of macaque J02185 fell below the detection limit at 2 weeks after peak viremia and remained low over the next 82 weeks (Fig. 1B). Macaque J02185 was still healthy at the end of the study and was classified as a non-progressor. Both animals maintained a detectable but gradually declining PBMC proviral DNA load throughout the course of the disease (Fig. 1C).

### Macaque sample selection and ultra-deep pyrosequencing of viral envelope

In order to gain a better picture of the envelope changes throughout disease progression, only animals that underwent a full 84 week course of observation were included for UDPS. For this purpose, PBMC samples from 5 time points of the progressor macaque K03135 (2, 16, 44, 64 and 83 weeks post-inoculation) and non-progressor macaque J02185 (2, 16, 44, 65 and 84 weeks post-inoculation) were selected for UDPS. In addition, it was reported previously that there was an unexpected peak of viral load in the duodenum sample from K03135 at 16 weeks post-inoculation [16]. The cause for this sudden elevated viral load in the gut tissue was unclear. To investigate if there was any unique viral population residing within the gut tissue at this time point, additional gut tissue samples from both animals at 16 weeks post-inoculation were also included for UDPS. Lastly, the two animals (M04123 and L03165) that died due to unrelated causes during sampling procedures were excluded for analysis as it would be impossible to extrapolate the data from these animals to disease progression.

**Figure 2:**
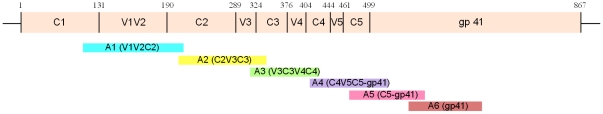
Schematic representation of the envelope regions covered by each amplicon (in reference to Env of the infectious molecular clone, SHIV-1157ipd3N4).

The current read length on the GS FLX Titanium series is approximately 400 bp. In order to cover the regions of interest in our study, we designed six overlapping PCR amplicons to capture the viral envelope regions (Fig. 2). Using this approach, we were able to sequence almost the entire gp120 and through the transmembrane domain of gp41. From the UDPS, we obtained a total of 574,225 reads with a median of 43,601 (range 13,964 to 81,666) reads from each sample. The UDPS data were cleaned to remove reads with PCR and UDPS artifacts while attempting to retain as many high-quality sequences as possible (see Materials and Methods). During the initial procedure, a median of 26% (range 23%–30%) reads were discarded from each sample. However, after the application of the AmpliconNoise algorithm, this number increased to a median of 43% (range 34%–47%). While this number may seem excessively high, there were still on average 18,000 reads per sample, ensuring sufficiently high coverage across the envelope to detect minor variants.

### Sequence diversity and divergence of intra-host viral populations

**Figure 3:**
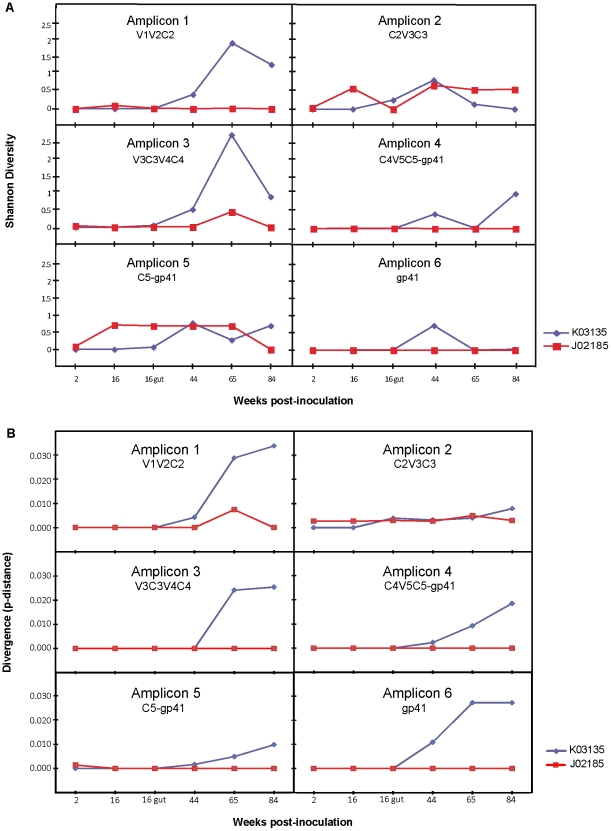
Phylogenetic analysis. (A) Shannon diversity. Longitudinal trends in sequence diversity analyzed using the Shannon Index. Time post-inoculation is shown on the x-axis and Shannon Diversity Index values are shown on the y-axis. All data shown are for AmpliconNoise processed amplicons. (B) Divergence. Longitudinal trends in sequence divergence from the time of inoculation. For direct comparison of sequences the mean Hamming distance at each time point was measured. Divergence was based on the Hamming distances to the SHIV-1157ipd3N4 inoculum for each amplicon. Time post-inoculation is shown on the x-axis and Hamming distance values are shown on the y-axis. Data for each amplicon are shown separately with macaque J02185 in red and macaque K03135 in blue, respectively.

Selective pressures imposed by the host immune system are the main driving forces shaping the evolutionary dynamics of the viral envelope. To examine the effect of immune selection on envelope evolution, we quantified the levels of diversity and divergence for each amplicon over time in both animals. The non-progressor macaque J02185 had a relatively low level of diversity in the entire envelope throughout the course of observation, with a maximum Shannon diversity index of only 0.72 in amplicon 5 beginning at 16 weeks post-inoculation (Fig. 3A). On the contrary, diversity for the progressor macaque K03135 began to increase at 44 weeks post-inoculation. It reached a peak of 2.05 in amplicon 1 and 2.71 in amplicon 3 by 64 weeks post-inoculation (Fig. 3A). The amplicons that showed a high level of diversity mainly comprised gp120. A relatively low level of diversity occurred within the gp41 in both animals. Similar to the levels of diversity, the non-progressor macaque J02185 had low divergence, showing that its viral populations did not deviate significantly from the inoculum strain over time, with a highest p-distance of only 0.007 in amplicon 1 at 65 weeks post-inoculation (Fig. 3B). The divergence of progressor macaque K03135 had a pattern similar to its diversity, with a progressively elevated p-distance from 44 weeks post-inoculation onwards. Amplicons 1 and 3 of the progressor macaque K03135 showed a high level of deviation from the inoculum strain, reaching a p-distance of 0.034 and 0.025, respectively, by 84 weeks post-inoculation (Fig. 3B). Additionally, the gp41 of K03135 developed a slightly higher divergence than the other envelope regions at 44 weeks post-inoculation (Fig. 3B). Mutations in this region might have evolved in response to the host immune pressure or selection of viral fitness, but there is no evidence or literature to support these possibilities. Lastly, the accumulation of minor mutations in gp41 of K03135 did increase its divergence despite a rather homogenous population over time as shown in the diversity. For the plasmid control, each of the six amplicons showed no diversity and a single population was present.

### Envelope evolution through disease progression

In order to further demonstrate the progressive nature of the diversification of envelope throughout disease progression, we aligned the amplicons from different time points with their corresponding reference sequence of the inoculum envelope. Furthermore, to highlight the similarities of some mutations observed in the infected pig-tailed macaque with other species, we included previous sequence data from a study involving the infected human and rhesus macaque for comparison [24]. In agreement with the diversity and divergence analysis, alignment of the amplicons from non-progressor macaque J02185 revealed extremely low variations within each time point and from the inoculum envelope (Fig. S1).

### C1 region

**Figure 4:**
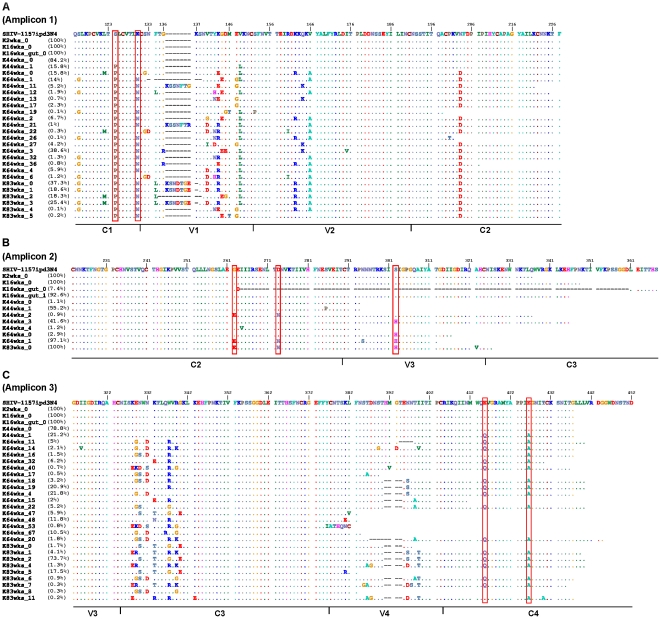
Amino acid alignments of the envelope from infected pig-tailed macaque K03135 as represented by “K”. (A) Amplicon 1, (B) Amplicon 2 and (C) Amplicon 3. Weeks post-inoculation is represented by “wks”. The value after “_” shows the population number. Deletions in the alignments are shown as “-”. The amount of the particular viral population at that time point is represented as percentage. Point mutations described in the text are highlighted by red color box.

**Figure 5:**
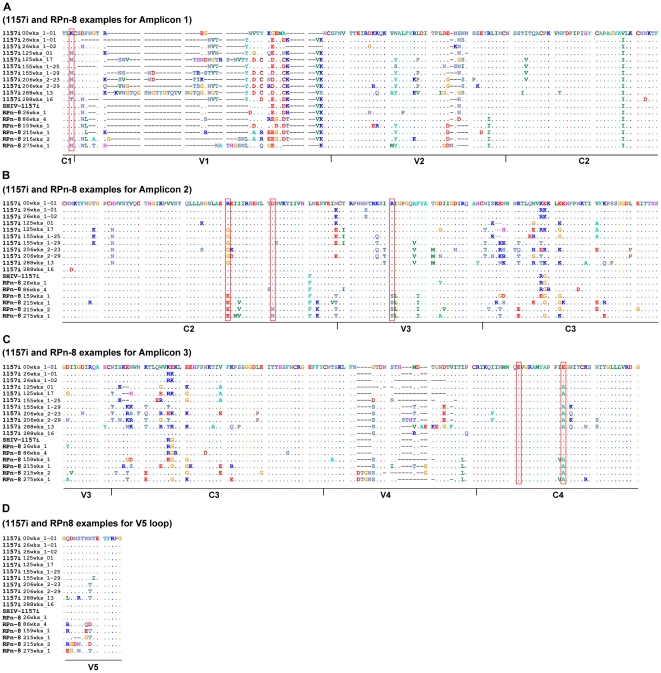
Amino acid alignments of the envelope from infected human (1157i) and rhesus macaque (RPn-8). 1157i and RPn-8 examples for (A) Amplicon 1, (B) Amplicon 2, (C) Amplicon 3 and (D) V5 loop. The inoculum for RPn-8 was SHIV-1157i (the initial infectious molecular clone). Weeks post-inoculation or infection is represented by “wks”. Sequences presented here are examples from each time point. These are not consensus sequences and do not represent all the sequence data from both the infected human and rhesus macaque. Point mutations described in the text are highlighted by red color box.

Contrary to J02185, substantially more mutations were observed in the progressor macaque K03135 as this animal progressed to AIDS. Among the mutations in K03135 was S124P, which evolved from being the minor population at 44 weeks post-inoculation to becoming the majority variant by 64 weeks post-inoculation (Fig. 4A). S124P is adjacent to the CD4 binding site and a proline mutation at this position might affect the CD4 binding site structure. Interestingly, the parental envelope of SHIV-1157ipd3N4 also contains a proline at position 124 [25]. Besides S124P, we also observed the emergence of K130N from 64 weeks post-inoculation onwards (Fig. 4A). K130N resulted in an additional PNGS and its appearance in the main viral population coincided with a consistently low CD4 count and the development of AIDS. More importantly, this identical mutation was also observed in the human and rhesus macaque during disease progression (Fig. 5A and Table 1) [24]. In addition, the same mutation also developed during disease progression in a rhesus macaque infected with a clade B SHIV [26].

**Table 1:** Comparison of HIV-1 clade C Env mutations between infected pig-tailed (K03135), rhesus macaque (RPn-8) and human (1157i).

Time points(post inoculation or infection)	Amino acid position 124 (C1)	Amino acid position 130 (C1)	Deletion (V1)	Insertion (V1)	Amino acid position 262 (C2)	Amino acid position 273 (C2)	Amino acid position 302 (V3)
**SHIV-1157ipd3N4**	S (100%)	K (100%)	0%	0%	G (100%)	D (100%)	S (100%)
**K03135 2 weeks**	S (100%)	K (100%)	0%	0%	G (100%)	D (100%)	S (100%)
**K03135 16 weeks**	S (100%)	K (100%)	0%	0%	G (100%)	D (100%)	S (100%)
**K03135 16 weeks gut**	S (100%)	K (100%)	0%	0%	G (100%)	D (92.6%)	S (92.6%)
**K03135 44 weeks**	S (84.2%)/P (15.8%)	K (100%)	0%	0%	G (99.1%)/E (0.9%)	D (99.1%)/N (0.9%)	H (41.6%)
**K03135 64 weeks**	P (100%)	K (19.5%)/N (80.5%)	14%	6.2%	G (2.9%)/E (97.1%)	D (2.9%)/N (97.1%)	H (100%)
**K03135 83 weeks**	P (100%)	N (100%)	18.3%	81.3%	E (100%)	N (100%)	H (100%)
**1157i 0 week**	–	K (100%)	0%	0%	R (96.8%)/W (3.2%)	D (100%)	R (100%)
**1157i 26 weeks**	–	K (82.7%)/N (6.8%)	37.9%	55.1%	R (100%)	D (100%)	R (100%)
**1157i 125 weeks**	–	K (19.2%)/N (46.1%)	0%	100%	R (26.9%)/G (73.1%)	D (100%)	R (100%)
**1157i 155 weeks**	–	K (13.6%)/N (81.8%)	9%	81.8%	R (4.5%)/G (95.5%)	D (100%)	R (100%)
**1157i 206 weeks**	–	N (100%)	0%	100%	G (100%)	D (100%)	R (100%)
**1157i 288 weeks**	–	K (15.6%)/N (18.7%)/T (65.7%)	0%	100%	R (81.2%)/G (18.8%)	D (100%)	R (65.6%)/K (31.2%)/G (3.1%)
**SHIV-1157i**	P (100%)	K (100%)	0%	0%	R (100%)	D (100%)	R (100%)
**RPn8 26 weeks**	–	K (100%)	0%	0%	R (100%)	D (100%)	R (100%)
**RPn8 86 weeks**	–	K (100%)	64.5%	0%	R (100%)	D (96.8%)/G (3.2%)	R (80.6%)/A (16.1%)/S (3.2%)
**RPn8 159 weeks**	–	K (100%)	3.3%	0%	R (23.3%)/G (26.7%)/E (50%)	D (100%)	S (100%)
**RPn8 215 weeks**	–	K (10%)/N (86.6%)	60%	33.3%	K (3.3%)/G (3.3%)/E (93.3%)	D (40%)/N (60%)	S (100%)
**RPn8 275 weeks**	–	N (88.8%)	0%	100%	E (100%)	D (2.8%)/N (97.2%)	S (100%)
**Notes:**	Adjacent to CD4 binding site	PNGS and charges	PNGS and charges	PNGS and charges		CD4 binding site	

Amino acid positions are in reference to the SHIV-1157ipd3N4 envelope.

Percentage of viral population containing a particular mutation is shown. “-”represent no information.

### V1V2 loops

**Figure 6:**
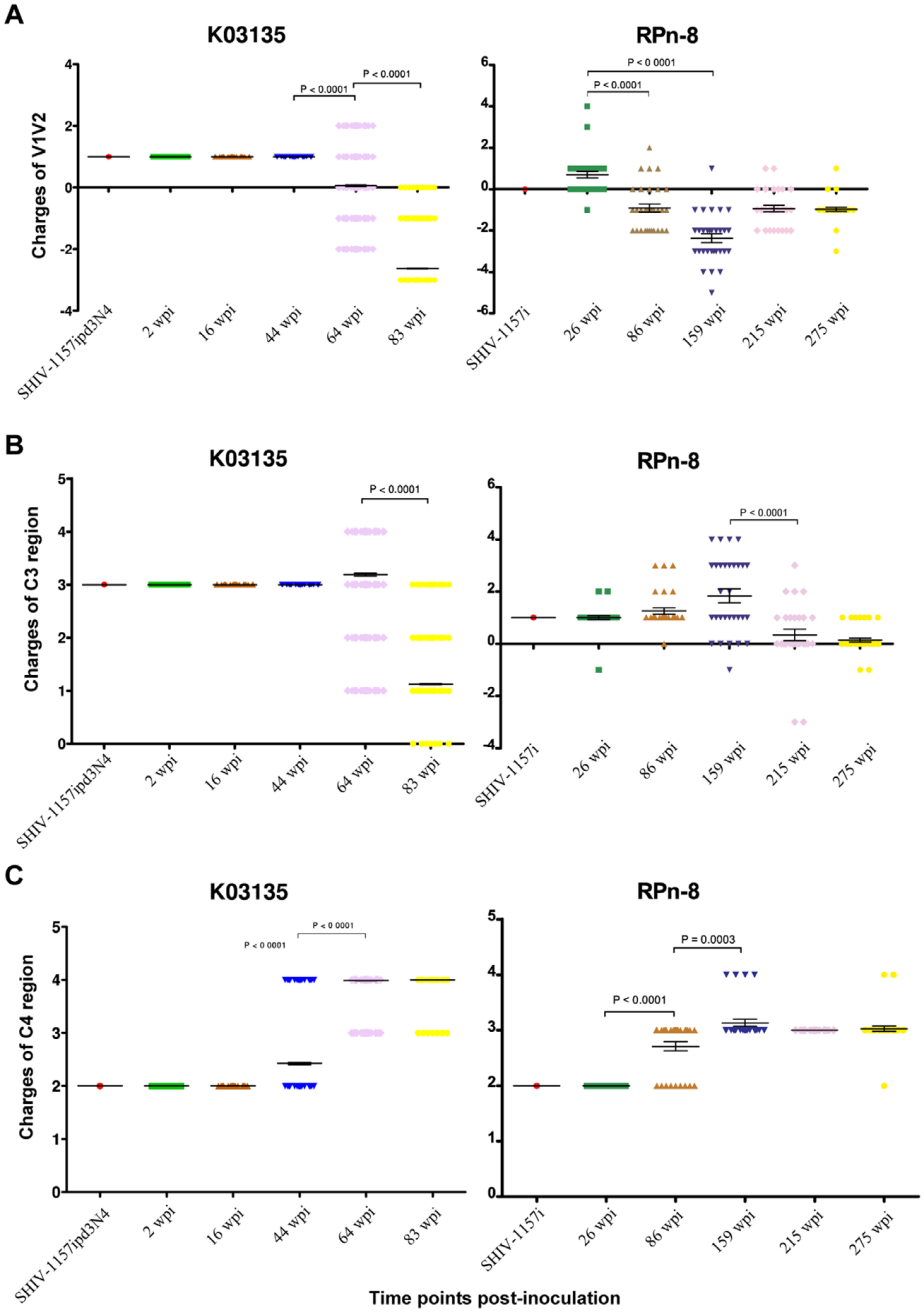
Comparison of envelope charges between the infected pig-tailed macaque (K03135) and rhesus macaque (RPn-8). (A) V1V2 regions, (B) C3 region and (C) C4 region. Time points are represented in weeks post-inoculation (wpi). The inoculum SHIV strain for K03135 was SHIV-1157ipd3N4 and SHIV-1157i for RPn-8. Each icon represents one envelope sequence.

14% and 6.2% of the viral populations contained deletions and insertions in the V1 loop, respectively, at 64 weeks post-inoculation (Fig. 4A). By 83 weeks, >80% of the viral populations contained an insertion in V1 but only 18.3% contained deletions. Deletions and insertions in the V1 loop are also common in human and rhesus macaque (Fig. 5A and Table 1) [24]. While insertions in the V1 loop usually involved PNGS, many of the mutations in the V1V2 region comprised of charged amino acids. The net charge of the V1V2 region for K03135 decreased significantly over time (P<0.0001) and was strikingly similar to those observed in the rhesus macaque (Fig. 6A). This charge reduction in V1V2 region was only observed as the animals progressed to AIDS.

### C2 region

Unlike the highly variable V1V2 region, only 3 non-transient mutations evolved within C2 region of Env. Among these mutations, G262E and D273N represent merely 0.9% of the viral populations at 44 weeks post-inoculation, but subsequently emerged as the dominant viral population by 64 weeks post-inoculation (Fig. 4B). These two mutations had also been observed in the rhesus macaque during disease progression (Fig. 5B, Table 1). Interestingly, D273N locates within the CD4 binding site and mutation at this position may affect envelope binding to CD4. More importantly, these mutations occurred only after the onset of AIDS in both the pig-tailed and rhesus macaques.

### V3 loop

The V3 loop of Env was relatively conserved in K03135, except for S302H that emerged at 44 weeks post-inoculation (Fig. 4B). Histidine at this position is extremely rare, 0 out of 756 sequences, in HIV-1 clade C envelope in the HIV sequence database. It is more commonly found in envelopes from other clades, such as A, B and D. So far, we had only observed 1 out of 18 HIV-1 clade C-infected patients from a Zambian cohort with histidine at this position (unpublished data). Since small changes in this region of the V3 loop could disturb the stability of the envelope trimeric structure of and affect CCR5 binding, mutations at this position could be important [27], [28]. Surprisingly, we detected a minor population of 7.4% in the gut tissue of K03135 at 16 weeks post-inoculation that contained a large deletion comprising the regions of C2V3C3 (Fig. 4B). It was determined that this was not as result of PCR or UDPS, given that it has passed our strict quality control procedure and contained an intact open reading frame. At this point, it is not clear whether this viral population is functional. However, it was reported previously that an envelope with truncation in V3 can still be functional [29], [30]. In addition, given that this population was unique to the gut tissue, it might serve as another example of HIV-1 compartmentalization [31].

### C3 region

The high degree of genetic polymorphism in the α2-helix of C3 had been noted previously in several studies involving human and rhesus macaque (Fig. 5C) [24], [32], [33]. The identical region is also highly variable in K03135 from 64 weeks post-inoculation onwards (Fig. 4C). Similar to the V1V2 region, mutations in this region frequently involved charged amino acids. A comparison of the net charge of C3 revealed a close similarity between the pig-tailed and rhesus macaques, with a significant (P<0.0001) decrease in the C3 charge occurring near the late stage of disease (Fig. 6B).

### V4 loop

85% of the viral populations carried deletions in V4 by 64 weeks post-inoculation (Fig. 4C). Deletions in V4 had been reported in pig-tailed macaque infected with a different SHIV [34]. In our study, the V4 deletions focused on the methionine located at the tip of V4, which was also progressively eliminated over time in the precursor envelope sequences of SHIV-1157ipd3N4 (Fig. 5C, Table 2) [24]. Surprisingly, deletions in V4 were presented at 16.5% of the viral populations from the non-progressor macaque J02185 at 65 weeks post-inoculation (Fig. S1).

**Table 2:** Comparison of HIV-1 clade C Env mutations between infected pig-tailed (K03135), rhesus macaque (RPn-8) and human (1157i).

Time points(post inoculation or infection)	Deletion(C2V3C3)	α2-helix(C3)	Deletion(V4)	Amino acid position 415(C4)	Amino acid position 426(C4)	Deletion(V5)	Deletion(gp41)
**SHIV-1157ipd3N4**	0%	Non variable	0%	E (100%)	E (100%)	0%	0%
**K03135 2 weeks**	0%	Non variable	0%	E (100%)	E (100%)	0%	0%
**K03135 16 weeks**	0%	Non variable	0%	E (100%)	E (100%)	0%	0%
**K03135 16 weeks gut**	7.4%	Non variable	0%	E (100%)	E (100%)	0%	1.2%
**K03135 44 weeks**	0%	Non variable	0%	E (78.8%)/Q (21.2%)	E (86.3%)/A (13.7%)	0%	0%
**K03135 64 weeks**	0%	Variable	85%	E (2.3%)/Q (97.7%)	A (100%)	0%	0%
**K03135 83 weeks**	0%	Variable	100%	Q (100%)	A (100%)	91.6%	0%
**1157i 0 week**	0%	Non variable	0%	E (100%)	E (100%)	0%	–
**1157i 26 weeks**	0%	Non variable	37.9%	E (96.6%)/G (3.4%)	E (100%)	0%	–
**1157i 125 weeks**	0%	Variable	46.2%	E (100%)	E (26.9%)/A (73.1%)	0%	–
**1157i 155 weeks**	0%	Variable	95.5%	E (100%)	E (59.1%)/A (40.9%)	0%	–
**1157i 206 weeks**	0%	Variable	84.6%	E (100%)	E (15.4%)/A (84.6%)	23.1%	–
**1157i 288 weeks**	0%	Variable	12.5%	E (100%)	E (59.4%)/A (40.6%)	15.6%	–
**SHIV-1157i**	0%	Non variable	0%	E (100%)	E (100%)	0%	–
**RPn8 26 weeks**	0%	Non variable	0%	E (100%)	E (100%)	0%	–
**RPn8 86 weeks**	0%	Non variable	0%	E (100%)	E (29%)/A (71%)	0%	–
**RPn8 159 weeks**	0%	Variable	60.0%	E (100%)	A (100%)	6.7%	–
**RPn8 215 weeks**	0%	Variable	10.0%	E (100%)	A (100%)	83.3%	–
**RPn8 275 weeks**	0%	Variable	88.9%	E (100%)	A (100%)	25.0%	–
**Notes:**		PNGS and charges	PNGS	CD4 binding site	CCR5 binding site	PNGS and charges	Heptad Repeat 1

Amino acid positions are in reference to the SHIV-1157ipd3N4 envelope.

Percentage of viral population containing a particular mutation is shown. “-”represent no information.

### C4 region

Despite a high sequence homology in the C4 region of Env, E415Q and E426A in K03135 emerged from 64 weeks post-inoculation onwards (Fig. 4C). E415Q is a significant mutation since this position is involved in CD4 binding. Surprisingly, the identical position was also mutated to glutamine over time in rhesus macaque infected with another SHIV [26]. Similarly, E426A, a CCR5 binding site, was observed in pig-tailed macaques, rhesus macaques and human as the disease progressed (Fig. 5C, Table 2). Since glutamic acid is a negatively charged amino acid, mutations at these two positions increased the overall charge of C4 (P<0.0001) as the disease progressed in K03135 (Fig. 6C). In addition, this pattern of increasing the charge of C4 resembled that observed in rhesus macaque during disease progression (Fig. 6C).

### V5 loop and gp41 region

**Figure 7:**
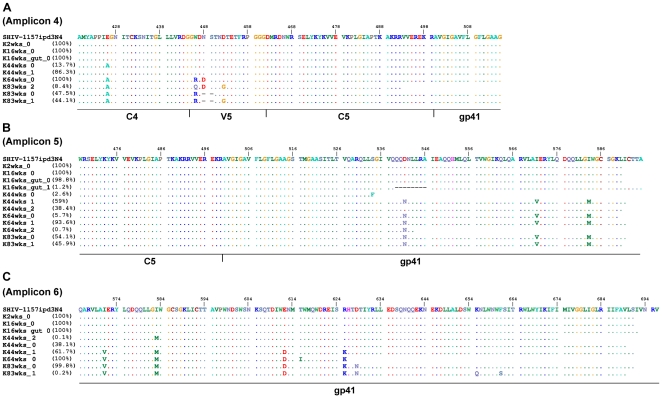
Amino acid alignments of the envelope from infected pig-tailed macaque K03135 as represented by “K”. (A) Amplicon 4, (B) Amplicon 5 and (C) Amplicon 6. Weeks post-inoculation is represented by “wks”. The value after “_” shows the population number. Deletions in the alignments are shown as “-”. The amount of the particular viral population at that time point is represented as percentage.

91.6% of the viral populations in K03135 contained deletions in V5 at 83 weeks post-inoculation (Fig. 7A). Similar deletions were also observed in rhesus macaque and human over time (Fig. 5D, Table 2). Lastly, the genetic variability of the envelope decreased sharply after the V5 loop. We did not find any mutations in the C5 region of the envelope and only few mutations were detected in gp41 (Fig. 7B and C). However, 1.2% of the viral populations in the gut tissue of K03135 contained deletions in the heptad repeat 1 of gp41 (Fig. 7C). Given that this deletion happened at such low frequency and localized in the gut tissue only, the impact of this mutation had on the overall disease progression could be minimal.

## Discussion

In this study, we provided a comprehensive view of the HIV-1 clade C envelope diversifications during disease progression between progressor and non-progressor pig-tailed macaques. Despite an extremely low level of diversity and divergence from the inoculum, we were still able to detect minor envelope variants in the non-progressor macaque J02185 over time, demonstrating the value of UDPS. However, there was clearly a mechanism by which J02185 was able to keep its infection well controlled. The fact that J02185 had envelope binding antibodies but no neutralizing antibodies against SHIV-1157ipd3N4, as reported in the previous study, indicated that neutralizing antibody responses are not the main mechanism behind its control over the virus [16]. In addition, the gradual reduction of PBMC proviral load occurred in J02185 without any significant decreases in its CD4^+^ T-cell counts, suggesting that this reduction was not due to a lack of target cells. This differs from the observation in the progressor macaque K03135, whose reduction of PBMC proviral load coincided with low CD4^+^ T-cell counts. Together, the data strongly suggests that cell-mediated immunity, such as cytotoxic T lymphocyte (CTL) responses, may be responsible for suppressing viral replication in J02185. However, due to a lack of information on the MHC class and CTL response analysis of J02185, we can only postulate that this was the main mechanism for viral control in this animal, which led to a reduction in virus production and the number of infected cells. Since mutations occur more frequently during viral replication, a diminished virus production will minimize the level of viral genetic variation, thus explaining the lack of envelope diversity and divergence in J02185 during the course of observation.

On the contrary, our data showed a very different picture for the progressor macaque K03135. The high plasma viral RNA load, PBMC proviral DNA load and decreasing CD4^+^ T-cell counts before the development of AIDS, indicated a lack of viral control by K03135 host responses. Similar to J02185, there were envelope binding antibodies but no neutralizing antibodies against SHIV-1157ipd3N4 present in K03135 [16]. Given this lack of neutralizing antibody responses, there may have been a lack of selective pressure resulting in rapid envelope evolution during the early phase of infection, thus explaining the low level of envelope diversity and divergence in K03135 before the development of AIDS. At the late stage of the disease, perhaps due to the combined effects of an ablated cellular immune system and natural selection for viral fitness, there was then a sharp increase in the envelope diversity and divergence in K03135. The notion that viral fitness was the main component for natural selection at the late stage of disease is supported by our observation that the distribution of envelope variations is not random; with mutations frequently occurring at specific regions or hotspots throughout the envelope that are proximal to important receptor binding sites over time, suggesting the presence of an active selection process.

In our study, we also observed that the envelope tended to follow a certain evolutionary pattern that correlated with disease progression. The majority of the envelope mutations were observed after the onset of AIDS. The most common mutations involved the addition of PNGS, which usually takes place in highly variable regions such as V1V2, α2-helix of C3, V4 and V5, and PNGS have been shown to play an important role in immune evasion [35], [36]. However, since the infected animals in our study had no neutralizing antibodies against the homologous virus, we believe these mutations evolved in response to the presence of high levels of non-neutralizing antibodies [16]. Although non-neutralizing antibodies cannot inhibit viral infection directly, they might still exert a selective pressure on the viral envelope through antibody-dependent cell-mediated cytotoxicity (ADCC), as suggested recently during SIV infection [37].

Changes in the length of envelope had been associated with immune escape and disease progression [1], [38]. Our data show that V1 accounted for the largest increase in length over time, and coincided with late stages of disease in the infected animals. There is a close relationship between the higher percentages of viral populations containing deletions in V4 and V5 with late stages of disease as well. Furthermore, our study observed a close association of changes in the envelope charges with disease progression. For example, the net charge in V1V2 and C3 tended to increase, while there was a decrease in C4 charges as the disease progressed. Variations in the charge of envelope had been suggested to affect viral fitness and were associated with disease progression [39]. The high number of charged amino acids in the α2-helix of C3 and its close proximity to the V4 loop suggest that it could be under heavy selective pressure from the non-neutralizing antibodies [33], [40], [41]. More importantly, charged amino acid mutations such as D273N, E415Q and E426A might affect the receptors binding ability of the envelope. Interestingly, similar mutations had been observed with clade A envelope as well [42].

In our study, we have demonstrated that the use of SHIV-1157ipd3N4 in pig-tailed macaque model can mimic the primary HIV-1 infection and disease progression in the human. Due to samples availability, we only analyzed PBMC vDNA. While plasma vRNA is derived from actively replicating virus population, infected PBMC contain incoming or newly synthesized vRNA in addition to integrated and unintegrated vDNA. Thus, studying PBMC allows us to document the complete HIV quasispecies repertoire which consists of actively replicating and archived viruses. Moreover, the use of UDPS in this study allowed us to detect and quantify minority variants that would have gone unnoticed using conventional sequencing technology. However, a major challenge with UDPS is its inherent errors that arise during the pyrosequencing process and those introduced by PCR amplification. Such errors are typically localized to homopolymers [43]. Therefore, careful filtering and control is essential to minimize the possibility of erroneous sequences. We implemented a carefully designed clean-up strategy to minimize the impact of 454 sequencing errors on interpreting our data. Our denoising approach removed most pyrosequencing errors due to sporadic base changes introduced during PCR while attempting to retain true biological sequence variation. The success of this approach is measured by our control plasmid amplicons as they were reduced to a single variant population.

In summary, UDPS has shown a strong association between the mutational dynamics of envelope and disease progression in the infected animals. In addition, we found a temporal relationship between the numbers of mutations occurring with little genetic variation before AIDS and more genetic variation afterwards. Such changes are likely due to a lack of immune surveillance in the early phase and selection for better viral fitness at the late stage of disease. Additionally, our data suggest that although non-neutralizing antibodies cannot inhibit the virus directly, they might still contribute to the evolution of envelope. More importantly, a majority of the mutations and evolutionary patterns of the envelope witnessed in SHIV-1157ipd3N4-infected pig-tailed macaques were also common in infected rhesus macaques and human, thus, suggesting a common selection pathway for the virus irrespective of the species studied.

## Materials and Methods

### Animals and viral stocks

All animals used in this study were housed and cared for according to the *Guide for the Care and Use of Laboratory Animals* at the Washington National Primate Research Center (WaNPRC), an Association for Assessment and Accreditation of Laboratory Animal Care International accredited institution. The animal quarters are maintained at 75–78°F with controlled air humidity and quality. The home cages of the animals are steam cleaned bimonthly and the waste pans are cleaned daily. Commercial monkey chow is fed to the animals once daily and drinking water is available at all times. Daily examination and any medical care of the animals are provided by the veterinary staff of WaNPRC in consultation with the clinical veterinarian. The experimental procedures were approved by the Institutional Animal Care and Use Committee (2370-20) at the University of Washington and conducted in compliance with the Public Health Services Policy on Humane Care and Use of Laboratory Animals (http://grants.nih.gov/grants/olaw/references/PHSPolicyLabAnimals.pdf). The animals were kept under deep sedation during all procedures with ketamine HCl at the dose of 10–15 mg/kg intramuscularly to alleviate any pain and discomfort. The animals were monitored by the Animal Technician or Veterinary Technologist while under sedation.

The construction of the infectious molecular clone, SHIV-1157ipd3N4, and the preparation of the viral stock were described previously [4]. All animal procedures and immunological analysis have also been published [16]. Briefly, four juvenile pig-tailed macaques were inoculated with SHIV-1157ipd3N4 intrarectally. Infected animals were monitored over a period of 84 weeks post-inoculation. Peripheral blood mononuclear cell (PBMC) and tissue samples were collected from the infected animals periodically.

### PCR amplification and amplicon library preparation for UDPS

Genomic DNA from PBMC and gut tissue samples was extracted following standard protocols. For amplicon library preparation, the full envelope was amplified from the samples with first round PCR primers positioned outside the envelope gene. The envelope from each sample was further amplified into 6 amplicons with six pairs of primers during the second round PCR. The envelope regions amplified by these primer pairs were V1V2C2 by primers env1, C2V3C3 by primers env2, V3C3V4C4 by primers env3, C4V5C5-gp41 by primers env4, C5-gp41 by primers env5 and gp41 by primers env6. Each sample was barcoded with a specific 10-nucleotides multiplex identifier (MID) and only primers containing the forward adaptor sequences were barcoded (Table 3). Two additional primers, env3_F_control and env3_R_control, were designed to amplify the envelope of an infectious HIV-1 clade C construct (1084ic) which served as a control [44]. Primer env1_F was modified to env1_F_a for amplifying the 65 and 84 weeks post-inoculation samples from J02185. Sequences of these primers are presented in Table 3. The PCR conditions used for amplicon amplification were 1 cycle of 95°C for 2 min, 35 cycles of 95°C for 30 sec, 60°C for 30 sec and 72°C for 30 sec and a final extension of 72°C for 4 min. All PCR was carried out with the FastStart High Fidelity PCR system (Roche, Indianapolis, IN). PCR products were isolated with the E.Z.N.A. Gel Extraction Kit (Omega Bio-Tech, Norcross, GA) and purified by the Agencourt AMPure magnetic beads (Beckman Coulter Genomics, Danvers, MA) following the conditions recommended for the Titanium amplicon library preparation (Roche/454 Life Sciences, Branford, CT). Purified amplicons were quantitated using the Quant-iT PicoGreen assay kit (Invitrogen, Carlsbad, CA) and pooled in equimolar concentration according to the manufacturer's recommendations. The pooled amplicons were then processed and sequenced on a Genome Sequencer FLX (Roche/454 Life Sciences, Branford, CT) at the Environmental Genomics Core facility (Engencore), Innovista Research District, University of South Carolina, Columbia, SC.

**Table 3:** Primers used for ultradeep-pyrosequencing library preparation.

Primer	Sequence (5′-3′)^a^	Genome location
env1_F	**cgtatcgcctccctcgcgccatcag**-MID-GATGCATGAGGATATAATCAGTTTATGGGA	309–338^b^
env1_F_a	**cgtatcgcctccctcgcgccatcag**-MID-CCGGGAAGAGTAATGTTACCTACAAAGGG	404–432^b^
env1_R	**ctatgcgccttgccagcccgctcag-**ACATTATGGCATGGTCCTGTCCCA	684–707^b^
env2_F	**cgtatcgcctccctcgcgccatcag**-MID-CCTGCTGGTTATGCGATTCTAAA	640–662^b^
env2_R	**ctatgcgccttgccagcccgctcag-**CAATAGAAAAATTCTCCTCTACAATTAAA	1102–1130^b^
env3_F	**cgtatcgcctccctcgcgccatcag**-MID-ACCAGGACAAGCAATCTATGCCAC	912–935^b^
env3_R	**ctatgcgccttgccagcccgctcag-**TCCTCCAGGCCTGAATGTTTCTGT	1357–1380^b^
env3_F_control	**cgtatcgcctccctcgcgccatcag-**MID-ATGAGGATAGGACCAGGACAAGCA	892–915^c^
env3_R_control	**ctatgcgccttgccagcccgctcag**-TCCTCCCGGTCTGAATATCTCTGT	1321–1344^c^
env4_F	**cgtatcgcctccctcgcgccatcag**-MID-TAAACATGTGGCAGGAGGTAGGAC	1229–1252^b^
env4_R	**ctatgcgccttgccagcccgctcag-**ATTGATGCTGCGCCCATAGTGCT	1546–1568^b^
env5_F	**cgtatcgcctccctcgcgccatcag**-MID-TGGAGGAGGAGATATGAGGGACAA	1374–1397^b^
env5_R	**ctatgcgccttgccagcccgctcag-**TACTCCAACTGTCGTTCCAAGGCA	1787–1810^b^
env6_F	**cgtatcgcctccctcgcgccatcag**-MID-TGGGGCATTAAGCAGCTC	1675–1692^b^
env6_R	**ctatgcgccttgccagcccgctcag-**CGATAATGGTGAGTATCCCTGCCT	2089–2112^b^

a lower-case letters: 5′ extensions for forward and reverse adaptor sequences; MID: 10-bp multiplex identifier; capital letters: envelope specific sequences.

b In reference to the envelope of the infectious molecular clone SHIV-1157ipd3N4 (GenBank: DQ779174.1).

c In reference to the envelope of the HIV-1 isolate HIV1084i (GenBank: AY805330.1).

### UDPS bioinformatics analysis

The initial sequence reactions yielding 574,225 reads that were processed to ensure high quality reads to reduce the typical sequencing errors from 454/Roche pyrosequencing. The data were cleaned by a set of scripts including the following criteria: (i) a perfect match to both the barcode and forward primer, (ii) >100 bases in length, and (iii) no undetermined bases (N). The 454 reads were then separated into samples by amplicons. The flowgrams corresponding to these reads were extracted, truncated at the first noisy signal [45], and then filtered to remove any read where this occurred in the first half of the flowgram. We then applied the AmpliconNoise pipeline to these samples separately using default parameters for GSFLX Titanium data [46]. The flowgrams were clustered with the PyroNoise program to remove 454 sequencing errors [47]. The forward primer and barcodes were removed from the resulting sequences, prior to their truncation to 400 bp. These were clustered with the SeqNoise program to remove PCR errors [46]. Finally, the Perseus *de novo* chimera classifier was applied to screen the sequences for chimeric PCR products which were then removed, giving denoised chimera checked sequences that were used in the following analysis. Alignments were generated and manually inspected to ensure any remaining variants with frameshifts or stop codons were removed. After the automated cleanup, a small number of problematic sequences remained that were associated with indels in homopolymer tracts. We modified these errors by deleting the extra base or adding a missing base relative to the inoculum sequence. As a control for 454 sequencing errors, a plasmid containing a subtype C envelope, 1084ic, was amplified and processed in parallel to the samples.

### Sequence diversity and divergence of intra-host virus populations

Diversity of viral sequences for each time point within each monkey was calculated using the Shannon Diversity Index. as follows
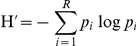
where H′ is the Shannon Diversity Index, R is the total number of species encountered and P_i_ represents the fraction of the entire population made up of species i [48]. Divergence of viral sequences for each time point within each monkey was calculated as the genetic distance between each sequence and the inoculum strain as calculated in MEGA v5.0 [49].

### Envelope charge analysis

The charge of the envelope was calculated with AminoTrack™ [50]. Briefly, arginine and lysine have a charge of +1, while aspartic acid and glutamic acid have a charge of −1. Statistical analysis was calculated using GraphPad Prism 5 (GraphPad Software, Inc., San Diego, CA).

## Supporting Information

Figure S1 **Amino acid alignments of the envelope from infected pig-tailed macaque J02185 as represented by “J”.** (A) Amplicon 1, (B) Amplicon 2, (C) Amplicon 3, (D) Amplicon 4, (E) Amplicon 5 and (F) Amplicon 6. Weeks post-inoculation is represented by “wks”. The value after “_” shows the population number. Deletions in the alignments are shown as “-”. The amount of the particular viral population at that time point is represented as percentage.
(TIF)
