# Supplementary figures and images for: Dynamics of Envelope Evolution in Clade C SHIV-Infected Pig-Tailed Macaques during Disease Progression Analyzed by Ultra-Deep Pyrosequencing
Source: PLoS One. 2012 Mar 12;7(3):e32827. doi: 10.1371/journal.pone.0032827 (PMC3299704; doi:10.1371/journal.pone.0032827)

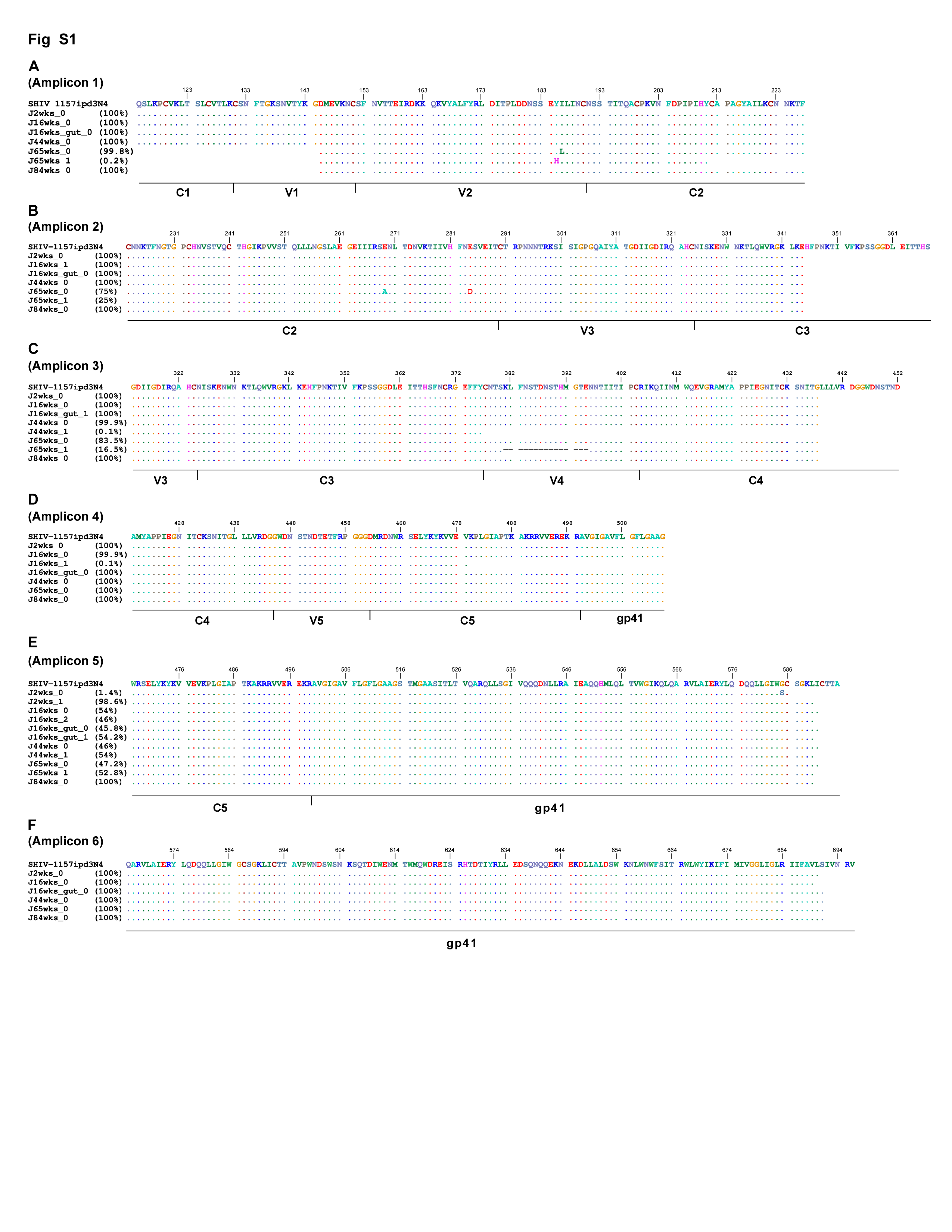

Supplement: Figure S1 — Amino acid alignments of the envelope from infected pig-tailed macaque J02185 as represented by “J”. (A) Amplicon 1, (B) Amplicon 2, (C) Amplicon 3, (D) Amplicon 4, (E) Amplicon 5 and (F) Amplicon 6. Weeks post-inoculation is represented by “wks”. The value after “_” shows the population number. Deletions in the alignments are shown as “-”. The amount of the particular viral population at that time point is represented as percentage. (TIF) [file pone.0032827.s001.tif]
